# *Lasius flavus* ants protect root aphid eggs from predators and pathogens during winter hibernation

**DOI:** 10.1098/rsos.250217

**Published:** 2025-07-09

**Authors:** Thomas Parmentier, Nicky Wybouw

**Affiliations:** ^1^ Département de Biologie des Organismes, Unit of Social Ecology, Université Libre de Bruxelles, Brussels, Belgium; ^2^ Department of Biology, Faculty of Sciences, Ghent University, Ghent, Belgium

**Keywords:** mutualism, trophobiont, Homoptera, cleaning behaviour, symbiosis, chemical signalling

## Abstract

Cooperative brood care is key to the ecology and evolution of social insects. Interestingly, social insects may also care for the brood of other species that dwell in their nests. This study explores how the yellow meadow ant *Lasius flavus* cares for the eggs of the root aphid *Anoecia zirnitsi* and how this service affects the resistance of aphid eggs to predators and pathogens. In winter, *A. zirnitsi* eggs were found exclusively in *L. flavus* nest chambers near the ant brood. Laboratory experiments showed that *L. flavus* detects, transports, piles and grooms the aphid eggs. We could recapitulate these caring behaviours in *L. flavus* using glass beads coated with chemical cues extracted from the aphid egg surface. Other ant species did not collect or nurse the eggs, suggesting a specific interaction between *L. flavus* and the eggs of *A. zirnitsi*. We further demonstrated that *L. flavus* strongly increased the aphid eggs’ protection against predators and fungal pathogens. Ants, however, were not essential for the eggs to hatch, and aphid nymphs were capable of independently colonizing grass roots. Our research highlights the crucial protection services *L. flavus* ants provide to root aphids in winter, while the potential costs and delayed benefits (honeydew provision) of this protection for the ants should be further explored.

## 1. Introduction

Different organisms provide ecological services to other species, such as pollination, dispersal, food provisioning and protection. These interactions can lead to mutualistic associations when the recipient also offers benefits in return [1]. However, not all relationships are reciprocal. In some cases, the recipient exploits costly services without providing anything in return, often through deception strategies, which shifts the relationship towards a more parasitic nature.

Ants play a crucial role in providing services to various organisms [2] and have advanced our understanding of the eco-evolutionary dynamics that drive the evolution of these services [3–5]. Protection of other organisms is the most widespread service provided by ants [2]. Ants defend other organisms, such as plants, aphids and caterpillars, by actively repelling threats from herbivorous and predatory arthropods through aggression, while also protecting against pathogens through hygienic behaviours such as grooming and secreting repellent substances [6,7]. In return, ants often receive nutritional benefits from these organisms, such as food bodies and nectar from plants, conidia from fungi or honeydew from aphids. The heterospecific protective and hygienic behaviours are a natural extension of the intimate social care ants show for the brood of their colony. The observed mutualistic interactions are often associated with specific morphological, behavioural, physiological and sensory adaptations in both partners [8]. However, ants are also exploited and deceived by other organisms, which manipulate them into providing costly services, such as protection, transport, food provisioning and brood care [9,10], offering little or no benefit in return. Certain species of myrmecophiles increase the costs to ants by feeding on their brood [10,11].

Different groups of ants and aphids have undergone strong coevolution, resulting in increasingly specialized and interdependent behaviour, morphology and chemical communication [12,13]. Various aspects of the often reciprocal services between ants and aphids on trees and herbs near ant nests have been relatively well studied [14]. However, less is known about the invisible interactions between ants and root-dwelling aphids located underground in or around the nest. Many of these aphid species, hereafter referred to as root aphids, live permanently on grass roots (monoecious), and often no winged females or males are known (anholocyclic = no sexual reproduction, only parthenogenesis) [15]. Root aphids are generally characterized by a close and intricate relationship with ants. Root aphids are protected, transported into safety by ant workers upon disturbance and possibly cleaned as well [16,17]. Root aphids, in turn, feed on the sap within the roots and secrete sugary honeydew, which probably serves as the primary food source for ant colonies [18]. Their hidden interactions with ants are so far only investigated by few studies, which are typically anecdotal and descriptive (but see [17,19–21]). Currently, the specificity of the interaction, whether, and how root aphids benefit from the presence of ants, and the cues that mediate these interactions are unknown. Furthermore, in temperate regions, almost all research on ant–aphid interactions is conducted during the growing season, while the winter months are hardly considered.

Interestingly, eggs of certain root aphid species are deposited underground, collected by ants, groomed and carefully piled near the ant brood at the onset of winter. Unfortunately, with a few exceptions, reports of ants providing nursing services to aphid eggs are extremely rare and limited to older faunistic notes [15,22–24] (but see recent photos by Matt Hamer in the UK [25]). This strategy is known for the root aphid species *Anoecia zirnitsi* and *Dyasaphis bonomii* (Palaearctic); *Anoecia setariae*, *Anoecia graminis* and *Anoecia oenotherae* (Nearctic) and *Protaphis middletonii* (Holarctic) [24,26]. For the species *Anoecia. pskovica*, there is discussion whether the eggs are collected by ants during hibernation (Zwölfer’s negative observations [26], Pontin’s positive observations [24]). *Anoecia krizusi* has also been reported by Pontin to have its eggs nursed, but these were probably misidentified *A. zirnitsi* individuals [27]. Most of the root aphids with ant-tended eggs thus belong to the genus *Anoecia*. Root aphids in this genus typically feed on the roots of different grasses (Poaceae), including cereals such as maize and barley, or sedges (Cyperaceae). *Anoecia* eggs are primarily collected by *Lasius* ant species, including *Lasius flavus* (Holarctic) and *Lasius neoniger* (North America) [24,26]. Studies report that *Lasius* ants may transport aphid nymphs hatching from the tended eggs and place them on the roots of their host plants [22,23], although Pontin states that nymphs in the Palaearctic species walk and search for host plants independently [24]. Ants do not only tend eggs of root feeding aphids, but may also care for the eggs of tree feeding aphids. This was demonstrated in a study on arboreal *Lasius productus* ants in Japan, which shelter eggs of the cypress feeding aphid *Stomaphis hirukawai* in galleries under bark or in nests at the base of cypress trees during winter. The study showed that grooming by *Lasius* ants protects the eggs of *Stomaphis* aphids from pathogenic fungi [21].

In this study, we tested different services provided by the yellow meadow ant *L. flavus* to the eggs of a root aphid. Colonies of the yellow meadow ant *L. flavus* (Fabricius, 1782) occur in high numbers in grassland habitats across the Palaearctic and construct underground nests, with or without a soil mound. *Lasius flavus* is well known for being subterranean, and workers rarely forage outside their nest. Their nests house different species of mutualistic root aphids in large numbers within chambers built around the roots of herbs and grasses [18,25,28–31]. Specifically, we assessed in this study: (i) the retrieval rate of aphid eggs, the nature of the attractive signal and whether this behaviour is specific to *L. flavus* ants; (ii) the role of *L. flavus* in protecting aphid eggs from soil predators; (iii) the role of *L. flavus* in protecting aphid eggs against pathogenic fungi; and (iv) the role of *L. flavus* in egg hatching and host plant colonization by the aphid nymphs.

## 2. Material and methods

### 2.1. Host ant and root aphid egg association

During the winter months (December–March) in the period 2014−2024, we found a total of 12 nests of *L. flavus* colonies with piles of black root aphid eggs (out of 30 opened nests) in a grass field (0.15 ha) in Ostend, Belgium (electronic supplementary material, figure S1). Laboratory experiments were conducted with eight *L. flavus* colonies, all of which stored root aphid eggs in their nests and were familiar with these eggs (table 1). During the winter period, we also found similar eggs in a nest of *L. flavus* on two occasions in a grass field in Ostend (51°12′5.84″ N, 2°55′29.25″ E) and in the neighbouring town of Middelkerke (51°10′32.80″ N, 2°47′41.78″ E).

**Table 1.** Overview of the ant nests used in the experiments.

ant species	nest	date	experiment	location	coordinates
*Lasius flavus*	LF_A	10/12/2014	retrieval	Ostend	51°12′18.00″ N, 2°55′17.55″ E
	LF_B	10/12/2014	retrieval	Ostend	51°12′18.39″ N, 2°55′16.94″ E
	LF_C	10/12/2014	retrieval	Ostend	51°12′18.13″ N, 2°55′16.88″ E
	LF_D	14/12/2014	fungus	Ostend	51°12′17.32″ N, 2°55′18.17″ E
	LF_E	14/12/2014	fungus	Ostend	51°12′17.19″ N, 2°55′18.54″ E
	LF_F	20/12/2022	identification	Ostend	51°12′18.32″ N, 2°55′17.30″ E
	LF_G	10/01/2024	predation	Ostend	51°12′16.96″ N, 2°55′18.33″ E
	LF_H	10/01/2024	hatching + identification	Ostend	51°12'17.77″ N, 2°55'17.86″ E
*Lasius niger*	LN_A	15/01/2015	retrieval	Ostend	51°12′18.31″ N, 2°55′17.45″ E
	LN_B	15/01/2015	retrieval	Ostend	51°12′17.64″ N, 2°55′16.17″ E
	LN_C	15/01/2015	retrieval	Ostend	51°12′16.34″ N, 2°55′19.46″ E
*Lasius fuliginosus*	LFul_A	27/03/2015	retrieval	Bruges	51°10′26.30″ N, 3°08′24.30″ E
	LFul_B	27/03/2015	retrieval	Bruges	51°10′13.6″ N, 3°09′33.9″ E
	LFul_C	30/03/2015	retrieval	Ostend	51°12′14.75″ N, 2°55′7.13″ E
*Formica fusca*	FF_A	27/03/2015	retrieval	Bruges	51°10′28.0″ N, 3°08′21.3″ E
	FF_B	27/03/2015	retrieval	Bruges	51°10′29.1″ N, 3°08′16.3″ E
	FF_C	27/03/2015	retrieval	Bruges	51°10′10.36″ N, 3° 9′25.30″ E
*Formica polyctena*	FP_A	27/03/2015	retrieval	Bruges	51°10′30.8″ N, 3°08′12.4″ E
	FP_B	27/03/2015	retrieval	Bruges	51°10′28.8″ N, 3°08′33.2″ E
	FP_C	27/03/2015	retrieval	Bruges	51°10′30.95″ N, 3° 8′23.83″ E
*Myrmica scabrinodis*	MS_A	27/01/2015	retrieval	Ostend	51°12′02.7″ N, 2°55′43.3″ E
	MS_B	27/01/2015	retrieval	Ostend	51°12′18.8″ N, 2°55′16.8″ E
	MS_C	27/01/2015	retrieval	Ostend	51°12′12.48″ N, 2°55′10.60″ E
*Tetramorium caespitum*	TC_A	18/01/2015	retrieval	Middelkerke	51° 9′27.87″ N, 2°44′53.71″ E
	TC_B	18/01/2015	retrieval	Middelkerke	51° 9′32.76″ N, 2°45′5.71″ E
	TC_C	18/01/2015	retrieval	Middelkerke	51° 9′28.27″ N, 2°44′55.79″ E

### 2.2. Species identification of the root aphid

*Molecular identification.* DNA was extracted from four root aphid eggs (nest LF_G) stored in 90% ethanol by adopting a previously established extraction protocol [32]. Following three rounds of washing for 1 min in 25 µl of sterile water, eggs were homogenized in 30 µl of PCR buffer (100 mM NaCl, 10 mM Tris-HCl, 1 mM EDTA, 3 mg ml^−1^ of proteinase K, set at pH 8). Egg homogenates were incubated for 40 min at 37°C. Proteinase K was inactivated by incubating the samples for 15 min at 95°C. DNA samples were diluted to their final concentration by adding 15 µl of sterile nuclease-free water. DNA was also extracted from an egg-laying adult root aphid (nest LF_H) in a 50 µl volume using the Quick-DNA Universal kit (BaseClear, The Netherlands). *COI* fragments were amplified using standard barcoding primers HCO2198 and LCO1490 [33]. PCR amplification was performed using DreamTaq DNA Polymerase (Life Technologies Europe BV) in a 50 μl reaction mixture with an annealing temperature of 50°C. Thirty cycles were run for all reactions. Two-way Sanger sequencing was performed for all egg and adult specimens (MACROGEN Europe BV). A 598 bp *COI* consensus sequence was established from the four egg samples and aligned with 13 reference *COI* sequences using MUSCLE (electronic supplementary material, table S1) [34]. For the *COI* nucleotide alignment, ModelFinder selected the TIM2+F+G4 model using the Bayesian information criterion [35]. A maximum-likelihood phylogeny was generated using IQ-TREE2 (v. 2.1.4, random seed number was set at 54 321) [36]. Ultrafast bootstrapping was performed with 10 000 replications [37].

**Figure 1.**
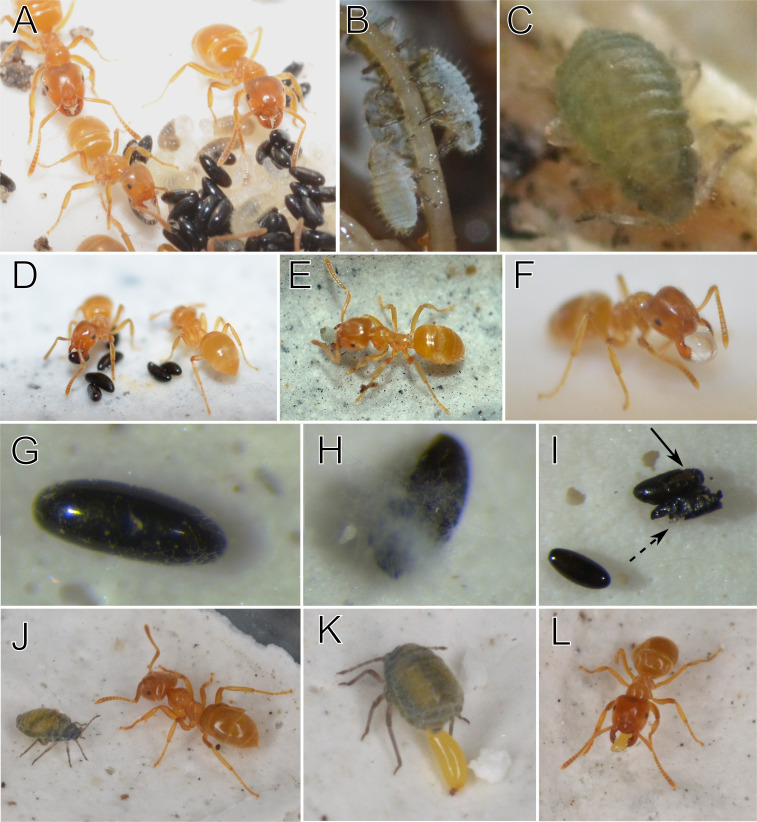
Overview of *Lasius flavus* ants and *Anoecia zirnitsi* aphids. (A) A pile of ant brood and black aphid eggs tended by *L. flavus* workers. Note that in field nests, aphid eggs are stored in separate piles close to the brood. (B) Newly emerged *A. zirnitsi* nymphs feeding on a grass root. (C) An *A. zirnitsi* nymph after a few moults. (D) Picking up of an aphid egg by a *L. flavus* worker. Note that workers often transport a cluster of aphid eggs rather than moving these eggs individually. (E) Transport of a newly emerged *A. zirnitsi* nymph. (F) Transport of a glass bead coated with an extract on the egg coating. (G) Egg with fungal hyphae at the onset of infestation. (H) Egg covered with fungus in a later stage. (I) One intact egg (bottom left), one egg that has been opened by the rove beetle *A. aeneicollis* (full arrow), and one egg that has been entirely damaged by the rove beetle predator (dashed arrow). (J) Ovipara of *A. zirnitsi* with yellow eggs visible in the abdomen and a *L. flavus* worker. (K) Ovipara of *A. zirnitsi* depositing an egg, one egg clearly visible in the abdomen. (L) The freshly laid egg is picked up by a *L. flavus* worker. Yellow eggs darken over time.

*Morphological identification.* A batch of root aphid eggs (approx. 100, nest LF_F) was placed at room temperature in an arena (diameter 9 cm) with moist plaster, 20 *L*. *flavus* workers and sprouting seeds of barley (*Hordeum vulgare*). After the nymphs started to feed on the roots of the barley seedlings, we regularly replaced the sprouting seeds. Aphid nymphs grew and underwent some moults (figure 1B,C), but we were unsuccessful in obtaining adults.

We collected three oviparous aphids on 3 November 2024 in nest LF_H where we had found a batch of eggs in January 2024. We morphologically identified these individuals after softening and clearing them using 10% KOH and acetic acid. We used a stereomicroscope (Leica MZ95, magnification 96×) and keys [27,38].

Voucher specimens of *L. flavus* workers and the associated eggs, nymphs and oviparae of *A. zirnitsi* are deposited at the Institute of Natural Sciences in Brussels (RBINS, IG 34991/001).

### 2.3. Transport of root aphid eggs and ant larvae by *Lasius flavus* and other ants

First, the retrieval rate of root aphid eggs and ant larvae was compared in *L. flavus* colonies. Ten root aphid eggs (length = 0.75 ± 0.04 mm, width = 0.31 ± 0.02 mm) were positioned in the centre of a Petri dish (90 mm) with a plaster bottom and were covered with a small lid. In each Petri dish, 15 workers were introduced and were allowed to acclimatize to the arena for 1 h. The trial started when the lid was lifted, allowing ants to interact with the eggs. The number of retrieved eggs was counted at 5, 10, 15, 20, 25, 30, 60 and 120 min. These trials were conducted with *L. flavus* colonies from three different nests (trials with root aphids: LF_A: *n =* 18, LF_B: *n =* 7, and LF_C: *n =* 7). Similarly, 10 first instar larvae (ant eggs are absent in *L. flavus* during winter, length larvae = 0.91 ± 0.01 mm, width larvae = 0.38 ± 0.02 mm) were placed in the centre of a Petri dish and covered with a lid for 1 h after adding 15 workers. Retrieval rate was assessed by counting the recovered larvae at the same time points as the trials with the root aphid eggs and using trials with the same nests (trials with larvae: LF_A: *n =* 18, LF_B: *n =* 7 and LF_C: *n =* 7; for details of nests see table 1). For each trial, different ant workers, larvae and root aphid eggs were used. Root aphid eggs and ant workers were isolated from the same nest in each trial. Next, similar trials and with counts taken at the same time intervals (5, 10, 15, 20, 25, 30, 60 and 120 min) with 15 workers of other ant species were conducted to assess whether these ants exhibit egg-retrieving behaviour. The following ant species were used: *Lasius niger*, *Lasius fuliginosus*, *Formica polyctena*, *Formica fusca, Myrmica scabrinodis* and *Tetramorium caespitum*. For each of these ant species, we conducted nine trials (three different nests with three trials/replicates). Control trials with their own larvae (three different nests with one trial) were also run for each of these ant species to check whether they show transport behaviour in this setup. Here, the numbers of retrieved larvae were only checked after 120 min, as we did not aim to measure detailed retrieval rates for these species.

For the retrieval trials with the *L. flavus* workers from nest LF_B and LF_C, we ran a mixed-effects Cox proportional hazards model (coxme package [39]) with the proportion of retrieved individuals (ant larvae or aphid eggs). Trial was modelled as a random factor. The proportional hazards assumption was met for these two analyses (proportional hazards test with the cox.zph function in package survival [40]). For the retrieval trials with the workers from nest LF_A, this assumption was not met. and therefore. we opted for another analysis. We compared the proportion of retrieved individuals (ant larvae versus aphid eggs) at 15 min after the start of the experiment using a binomial glmer, with trial as a random factor.

### 2.4. The nature of the cue triggering the transport of root aphid eggs by *Lasius flavus*

In a first series of experiments, we immersed the eggs in a solvent aiming at removing chemical cues that might elicit the ritualized picking up and transportation of aphid eggs. Ten root aphid eggs collected from LF_A were washed in 30 µl of apolar solvent hexane for 5 min and this was repeated three times. Washed root aphid eggs were placed into a Petri dish and offered to 15 workers from LF_A (following the protocol above), and egg retrieval was scored at the same time points. Similarly, the effect of washing root aphid eggs with the more polar solvent hexane/methanol (1 : 2, v/v) on their attraction was tested [41]. For each solvent, 10 trials were conducted. Unique eggs were used in each trial. As the proportional hazard assumption was not met, we compared the proportion of aphid eggs (untreated versus washed with hexane/methanol, and untreated versus washed with hexane) at 15 min after the start of the experiment using a binomial glmer, with trial as a random factor.

In a second series of experiments, extracts of the rinsing were placed on glass round beads (dummies) to test whether the chemical signal can be extracted and transferred. All glass beads were pre-cleaned with hexane and dried. Next, 20 root aphid eggs were extracted for 5 min in 30 µl hexane : methanol (1 : 2). The solvent with the extract was transferred to a glass vial with 10 pre-cleaned glass beads (0.5 mm diameter). We used the extract from two eggs per bead to ensure that a detectable concentration of attractive cues was transferred. When the solvent was evaporated, the glass beads were put in a Petri dish with 15 *L. flavus* workers. The behaviour of ants towards the beads and their retrieval was monitored for 30 min. This was repeated twice (one trial with nest LF_A and one trial with LF_B). Finally, we also offered 10 pre-cleaned glass beads without extract in a Petri dish with 15 *L. flavus* workers, to assess whether *L. flavus* ants retrieve beads without cues. This control was repeated seven times (four trials with workers from LF_A and three trials with workers from LF_B), so more than the two trials with root aphid egg extract as the beads were available in much greater numbers than the eggs. We compared the total number of glass beads with and without extract retrieved per trial and grouped per nest with a non-parametric aligned rank transform test using the package ArTool [42].

### 2.5. Protection of root aphid eggs by *Lasius flavus* against pathogens

We tested whether *L. flavus* clean the root aphid eggs and offer protection against pathogenic fungi. Throughout the following experiments, aphid eggs originated from the same nest as the used *L. flavus* workers. We placed five root aphid eggs in a Petri dish with a moist plaster bottom. The eggs were positioned within the central portion of the Petri dish without touching each other and within a radius of 0.5 cm from the centre. To promote infection by fungi that are naturally present in ant nests and surrounding soil material, 0.2 g of soil from an ant nest (LF_D) was taken. Soil was spread at the outer edge of the Petri dish. The central area of the Petri dish remained uncovered by soil, allowing eggs to rest directly on the plaster surface. Five trials with 30 *L. flavus* workers from nest LF_D were established and five trials without ant workers were used as controls. The same number of trials was tested with workers and the soil of nest LF_E. Petri dishes were stored at 4°C (median winter temperature in the study area) in darkness for two months. Following these winter-like, hibernation conditions, eggs were inspected for the presence of fungal hyphae. We used non-parametric Wilcoxon signed-rank tests to compare the mean number of eggs with hyphae in a trial with ants and without.

Subsequently, Petri dishes were placed at room temperature and the proportion of eggs that hatched with and without ants was compared after one month. Every week of the three month period of this experiment (two months in hibernation + one month at room temperature), dead ant workers were removed and replaced by workers of the corresponding colony stocks.

### 2.6. Protection of root aphid eggs by *Lasius flavus* against predators

We tested whether *L. flavus* protects root aphid eggs against predators. As a predator representative, we selected the rove beetle *Atheta aeneicollis* (Staphylinidae: Aleocharinae). *Atheta aeneicollis* is a common soil dweller that lives in the same habitat as *L. flavus*. It also falls, with its body length of 3.5−3.8 mm [43], within the same size range as *L. flavus* (2.2−4.8 mm) [44]. Rove beetles are known to feed on arthropod eggs [45,46]. Rove beetle individuals were collected by sieving compost material in the same area as the ants and root aphids (February 2024). For these experiments, all ants and eggs were collected from nest LF_G. Tests to compare the number of damaged aphid eggs by the beetle with and without ants were conducted in Petri dishes with a moist plaster bottom. A standardized pattern of eight furrows was carved into the plaster, which offers hiding places for the beetle from ant aggression during the tests (cf. [47]). First, five root aphid eggs were placed in the centre of each Petri dish. For the treatment with ants, five *L. flavus* workers of the host nest (LF_G) were added to the Petri dish. We used only five workers, compared to 30 in the pathogen resistance experiment, as preliminary tests showed that the beetles could not survive in the presence of a high number of workers. After 1 h, a beetle individual was added, both in the treatment with and without ants (control). The treatment with ants and the control was repeated eight times, with unique ants, aphid eggs and beetle individuals in each trial. Petri dishes were stored at 10°C and after 48 h, the number of eggs was counted and checked if there were any signs of damage and biting (magnification 64×). The higher temperature in this experiment compared with the experiment with fungi was to stimulate activity and predatory behaviour of the rove beetle, as the beetle was observed to be inactive at 4°C. We used a non-parametric Wilcoxon signed-rank test to compare the mean number of damaged eggs in a trial with ants and without.

### 2.7. Hatching of root aphid eggs

In this experiment, we investigated whether aphids require assistance from ants or are stimulated by ant interactions to emerge from their eggs. Here, we collected eggs that had been tended by ants from nest LF_H in controlled hibernation conditions. Once the first aphid nymph emerged at 4°C (4/4/24), indicating the eggs were close to hatching, we started the experiment. We placed 10 eggs into small Petri dishes filled with plaster (diameter 50 mm). This setup was repeated 10 times, with five trials having five *L. flavus* workers and five trials without ant presence (control). Petri dishes were placed at room temperature to simulate spring-like conditions and induce hatching. We used the setup where we tried to grow the aphid nymphs (a batch of root aphid eggs (approx. 100, nest LF_F) was placed at room temperature in an arena (diameter 9 cm) with moist plaster, 20 *L. flavus* workers and sprouting seeds of barley (*Hordeum vulgare*)) to check whether the nymphs found the roots independently or were carried to the roots by the ants. We observed for at least 2 h, whether the hatched nymphs were transported to the roots or independently walked to the roots.

We compared the time the eggs needed to hatch in the presence and absence of *L. flavus* with a mixed-effects Cox proportional hazards model (coxme package). Treatment (two levels: with ants versus without ants) was modelled as a fixed factor, trial (10 levels) as a random factor. The proportional hazards assumption was met (proportional hazards test with the cox.zph function in package survival [40], treatment: χ^2^ = 1.02, d.f. *=* 1, *p =* 0.31). The cumulative distributions of eggs hatched over time were plotted with the ggsurv function and were based on proportional hazards models without accounting for the random factor trial.

## 3. Results

### 3.1. Species classification identifies *Anoecia zirnitsi* as the egg-laying root aphid

**Figure 2.**
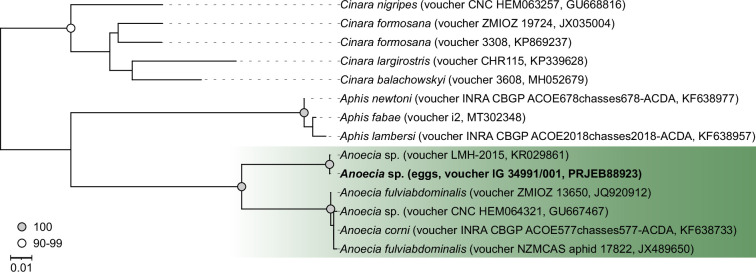
A mitochondrial marker places our root aphid species within the *Anoecia* genus. A maximum-likelihood phylogeny was generated based on a 598 bp *COI* fragment, isolated from root aphid eggs. Ultrafast bootstrap support values are depicted as coloured circles. The *Anoecia* genus is indicated by a green background. For each phylogenetic tip, voucher ID and NCBI accession number are provided in brackets.

Using an approximately 600 bp *COI* fragment, maximum-likelihood phylogenetic reconstruction revealed that the root aphid eggs were laid by an aphid species within the genus *Anoecia* (figure 2). The *COI* nucleotide sequence of the four aphid eggs exhibited an exact match with that of an unidentified specimen, *Anoecia* sp. 1 LMH-2015 (figure 2). We were unable to culture the nymphs emerging from the eggs to adulthood, a requirement to determine species identity morphologically. However, sequencing of a *COI* amplicon from an egg-laying adult root aphid (ovipara) collected in autumn in nest LF_H generated a reliable *COI* fragment of 348 bp from the trace data, exhibiting a 100% match with the larger *COI* fragment of the root aphid eggs. The COI sequence data are accessible at ENA (PRJEB88923, ERP171980). Consequently, we further resolved species identity of the eggs within the *Anoecia* genus by morphologically identifying the corresponding oviparae, using the keys and figures provided in [27,38], although the morphological description of oviparae is inadequate. Morphological distinct characters were the greenish colour, the presence of large marginal tubercles on the prothorax, indistinct siphunculi, the lack of dorsal sclerotization, eyes with few ommatidia, finger-form processus terminalis and the relative length of the third antennal segment. Within the genus *Anoecia,* these characters are unique for *A. zirnitsi.* The presence of eggs in a nest of an ant colony (within the Palaearctic *Anoecia,* only *A. zirnitsi*, *A. krizusi* and *A. pskovica* are known to display this behaviour), the shape of the eggs (eggs of *A. pskovica* are more spherical [24]) and the host plants (*A. pskovica:* Cyperaceae, but only Poaceae near the nests of the colonies in this study) further support *A. zirnitsi* as the egg-laying aphid species in this study.

*Anoecia zirnitsi* has a monoecious, holocyclic life cycle. The fundatrix emerges from the eggs in spring, followed by several generations of parthenogenetic females. In autumn, oviparous females are produced, which lay overwintering eggs in *L. flavus* nests. Zwölfer did not find males in 29 populations of *A. zirnitsi*, leading to the suggestion that the eggs might be parthenogenetic, a phenomenon considered unique among aphids [26]. However, Paul later reported the discovery of a male, indicating that eggs may be produced sexually, at least in some populations [27]. Two of the three collected *A. zirnitsi* oviparae were isolated with *five L. flavus* ants of nest LF_H in a Petri dish (diameter 4.5 cm) with a plaster bottom. Large yellow eggs were clearly visible in the body of these oviparae (figure 1J). The oviparae started to lay these yellow eggs the following day (figure 1K). One ovipara deposited three eggs, the other four. The eggs were rapidly collected by the workers (first egg within a minute) and piled (figure 1L). Since all aphid eggs of this study were found in nests within a single small grass field (0.15 ha; table 1), and the nymphs (figure 1C) hatching from eggs collected from other nests in this field appear identical, we assume all specimens belonged to *A. zirnitsi*.

### 3.2. Only *Lasius flavus* carries *Anoecia zirnitsi* eggs to safety from the ant panel

**Figure 3.**
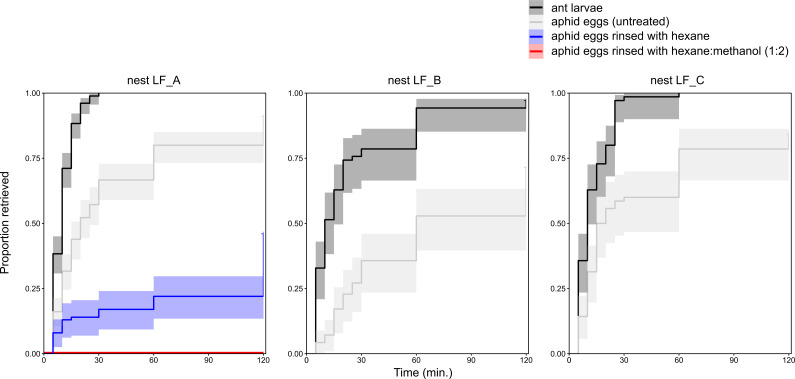
Comparative retrieval rate (= proportion carried into safety) of untreated root aphid eggs, root aphid eggs rinsed with solvent and first instar ant larvae by three nests of *Lasius flavus* (LF_A, LF_B and LF_C). Left panel: nest LF_A: retrieval rate of ant larvae, untreated root aphid eggs, root aphid eggs rinsed with hexane and root aphid eggs rinsed with methanol : hexane (2 : 1). Middle panel and right panel: retrieval rate of ant larvae and untreated root aphid eggs by nest LF_B and LF_C, respectively. The 95% confidence intervals (CIs) are represented by bands.

In the study field, where we found *A. zirnitsi* eggs in 12 of the 30 nests of *L. flavus* colonies, neither *L. niger* (*n* = 15) nor *M. scabrinodis* (*n =* 10) nests contained any of these aphid eggs. When detecting a root aphid egg in the middle of an arena, *L. flavus* workers showed a characteristic behaviour. The workers started to antennate, opened their mandibles and picked up a single egg gently between their mandibles in the longitudinal direction (figure 1D; electronic supplementary material, video S2). Typically, the workers quickly ran to the edge of the arena and dropped the eggs near other already retrieved eggs. In some cases, workers carried the eggs for a longer time around, before dropping them near other eggs at the edge of the arena. When disturbing a natural *L. flavus* colony, workers often grabbed a pile with multiple eggs (electronic supplementary material, video S3). Although root aphid eggs were quickly collected by *L. flavus*, their own first instar larvae were rescued at a faster rate with workers of all three tested nests (nest LF_B: Cox mixed-effects model: LR test: χ^2^ = 46.4, d.f. *=* 1, *p* < 0.001; col. LF_C: Cox mixed-effects model: LR test: χ^2^ = 91.4, d.f. *=* 1, *p* < 0.001; col. LF_A: mixed binomial model: χ^2^ = 70.4, d.f. *=* 1, *p* < 0.001; figure 3). The other six ant species did not show interest in the root aphid eggs, simply passing or ignoring them. For these six ant species, all trials with the aphid eggs, therefore, yielded a value of 0 at the 120-time point under the ‘status’ column in the dataset ‘retrieval’. In rare cases eggs were picked up, but then ripped apart or dropped a bit later without being taken care of. Note that these ants transported their own larvae into safety, showing that all six ant species are capable of rescue behaviour within the experimental setting. For these six species, all trials with their larvae yielded a value of 1 at the 120-time point under the ‘status’ column in the dataset ‘retrieval’.

### 3.3. A chemical cue on *Anoecia zirnitsi* eggs triggers the care-taking behaviour of *Lasius flavus*

A smaller proportion of root aphid eggs treated with hexane were retrieved than untreated eggs (nest LF_A: binomial glmer: χ^2^ = 18.56, d.f. = 1, *p* < 0.001, figure 3). When treated with methanol : hexane (2 : 1), eggs completely lost their attractivity and were left in the middle of the arena (figure 3). Glass beads coated with methanol : hexane (2 : 1) extract of the root aphid eggs were significantly more collected than control beads (aligned rank transform test, treatment: *F* = 484.4, *p* < 0.001). More specifically, control beads without extract were ignored and never picked up (0 of 10 beads retrieved in the fourth and third trials conducted with LF_A and LF_B, respectively). In contrast, *L. flavus* picked up a large part of beads coated with aphid egg extract over a period of 30 min, brought them into safety to the side of the arena, and tended them (figure 1F; electronic supplementary material, videos S4, 9 of 10 beads in single trial with nest LF_A and 4 of 10 beads in single trial with nest LF_B).

### 3.4. *Lasius flavus* protects root aphid eggs against fungal pathogens

Fungal hyphae were easily recognized in these treatments, showing the efficacy of the experimental setup (figure 1G,H). Ant presence significantly reduced the likelihood of fungal infection (nest LF_D: Wilcoxon rank sum test, *W* = 0, *p* = 0.006; nest LF_E: Wilcoxon rank sum test, *W* = 0, *p* = 0.004). The five eggs in each of the 10 arenas without ants were overgrown by fungi after two months. In contrast, the five eggs of nine of the 10 trials with *L. flavus* were shiny and no traces of hyphae could be detected. Aphid eggs were clearly tended by the ants in a small pile of five. When exposing the arena to light, ants readily carried the eggs away. In one trial with ants (LF_D), four out of five eggs were covered with hyphae. These eggs were not nursed. Petri dishes were then placed at room temperature. Over the course of one month, all but one egg (44/45) of the nine trials with ants hatched. The five eggs of the trial, where hyphae were found in the presence of ants, did not hatch, but were completely overgrown by fungi at room temperature. The eggs that were kept without ants were already overgrown by fungi after two months in the fridge and none of them hatched in the next weeks at room temperature.

### 3.5. *Lasius flavus* protects root aphid eggs against a predatory rove beetle

The predatory rove beetles survived (and were unharmed) in all trials with and without ants. The ant workers showed aggression when the beetle approached, but due to hiding opportunities and a low number of ants, beetles could easily continue to evade encounters with the ants. In the treatment with ants, aphid eggs were collected and stored in piles. A large part of the eggs was clearly damaged by the beetle in the absence of ants (the mean number of damaged eggs: 1.88 ± 1.36 s.d.), whereas all eggs were consistently intact and healthy in the eight trials with ants (Wilcoxon rank sum test to compare mean number of damaged eggs, *W* = 4, *p =* 0.001). Damaged eggs by *A. aeneicollis* were characterized by bite marks and a shrivelled appearance. Eggs could also be torn apart with their contents consumed (figure 1I). We never observed *L. flavus* workers preying on the root aphid eggs throughout all our experiments.

### 3.6. *Lasius flavus* is not essential for egg hatching and host plant attachment of *Anoecia zirnitsi*

Forty-one out of 50 eggs hatched with ants, whereas 40 out of 50 eggs hatched without ants after 30 days (electronic supplementary material, video S3). The timing of egg hatching was also very similar (Cox mixed-effects model: LR test: χ^2^ = 0.41, d.f. *=* 1, *p* = 0.52). We did not observe the ants transporting the hatched nymphs to the roots; instead, the nymphs colonized the roots independently.

## 4. Discussion

Brood care is a vital task in social insect colonies, involving storage in piles, careful cleaning, protection and swift relocation in response to disturbance [48]. However, to what extent brood care extends to ant-associated insect species that have an underground lifestyle and during hibernation periods remains poorly understood. Collectively, our research highlights that during the winter hibernation period, *L. flavus* ants exhibit heterospecific brood care of the root aphid *A. zirnitsi,* greatly benefiting the latter’s protection against fungal pathogens and predation.

Eggs of the root aphid *A. zirnitsi* were exclusively found within the underground nest chambers of *L. flavus* colonies in the winter months (electronic supplementary material, video S1). Prior faunistic studies similarly identified *L. flavus* as the specific ant host for these eggs during winter [24,26]. Lab experiments revealed that the aphid eggs only induced caring behaviour in *L. flavus*. Other ant taxa ignored the eggs or occasionally bit and damaged them, whereas *L. flavus* rapidly secured the eggs, stored them in piles and started to lick and clean the eggs. We found a high rate of egg transport by *L. flavus*. However, their own brood was still secured more rapidly. A previous study found that some adult root aphids are secured at the same rate as the brood [17]. Studies on symbiotic associations in social insects have shown that chemical cues from the symbiont can induce recognition and attraction by the host [49–52]. These cues may mimic the cuticular hydrocarbon profile of the ant host, or appeasement signals may also be produced [49–52]. Aphids have been reported to mimic the hydrocarbons of their ant host (workers [53]; larvae [13]), probably inducing amicable behaviour in the ants and facilitating adoption into the colony. We verified that the causal cue(s) were not hydrocarbons but consisted of non-volatile compound(s) with higher polarity. Unfortunately, we could not fully resolve the chemical identity.

The soil environment presents various threats to insects, with the primary dangers being predation by other invertebrates and infection by pathogenic bacteria or fungi. Ants protect their defenceless brood against predators by piling them together deep inside the nest [2]. Predators are usually unable to penetrate into the nest as the ants attack and deter them by biting, stinging or spraying formic acid. Only a small group of highly specialized arthropods succeed in breaking this defence line by deception and obtaining access to the ant brood [10,54]. Here, we showed that this defence line is also beneficial for the protection of root aphid eggs against predators. We found that a small number of ant workers were already highly efficient in keeping away potential egg predators. The threat of pathogenic bacteria and fungi, which occur in high abundances and diversity in the soil, is also challenging. Social insects protect their brood against these pathogens by licking them clean and transferring their saliva, which contains compounds with antimicrobial and antifungal properties, on the brood surfaces [55]. Ants may also smear secretions of the metapleural and poison glands for sanitation on the brood surface [6,56]. Root aphid eggs were also intensively groomed, and the ants’ hygienic services were indispensable for their survival. These results confirmed a previous study on the ant protection of *Stomaphis* tree aphid eggs from fungal infection [21]. Heterospecific cleaning services are widespread in different biomes [57], but are barely reported in soil systems.

Ants were not essential for aphid egg hatching. Newly emerged aphid nymphs were mobile (electronic supplementary material, video S3) and during several hours of observation in laboratory settings, they regularly colonized roots and started feeding without assistance from ant workers. This observation contrasts with some anecdotal reports that claim that some root aphid nymphs are carried as cattle to the roots by their host ants [22,23,58]. Transport of *A. zirnitsi* nymphs occurred, yet infrequently, upon disturbance in lab conditions (figure 1E). These nymphs were brought back to the ant brood pile, but we did not observe *L. flavus* placing the nymphs on the roots in our laboratory settings. Nonetheless, it remains possible that under natural, and dark conditions, root aphids can be transported by *L. flavus* to feeding sites. The aphid *A. zirnitsi* is reported to move outside nests of *L. flavus* colonies in spring. It is unclear whether they are always ant tended [26]. It is also likely that other ant species or competing *L. flavus* colonies visit and tend the *A. zirnitsi* populations when they are not strictly bound to the *L. flavus* nest where they were born. These scenarios suggest that *A. zirnitsi* root aphids may utilize *L. flavus* ants for protection of their eggs during winter hibernation without providing equivalent rewards to the host colony later in the year.

This research sheds light on the different services provided by the yellow meadow ant to the eggs of a root aphid. Future research should focus on how costly the nursing of root aphid eggs is for ants during winter. In addition, the ant–aphid interaction should be surveyed throughout the entire year to determine whether *L. flavus* nests receive delayed benefits from their potential nursing investment during winter through a consistent supply of honeydew from the aphids in the active season.

## Data Availability

The data files and R code to conduct the analyses are available at Dryad [59]. Supplementary material is available online [60].
